# Mutation Induced Conformational Changes in Genomic DNA from Cancerous K562 Cells Influence Drug-DNA Binding Modes

**DOI:** 10.1371/journal.pone.0084880

**Published:** 2014-01-08

**Authors:** Debjani Ghosh, Subrata Kumar Dey, Chabita Saha

**Affiliations:** School of Biotechnology and Biological Sciences, West Bengal University of Technology, Salt Lake, Kolkata, India; University of Quebect at Trois-Rivieres, Canada

## Abstract

Normal human genomic DNA (N-DNA) and mutated DNA (M-DNA) from K562 leukemic cells show different thermodynamic properties and binding affinities on interaction with anticancer drugs; adriamycin (ADR) and daunomycin (DNM). Isothermal calorimetric thermograms representing titration of ADR/DNM with N-DNA and M-DNA on analysis best fitted with sequential model of four and three events respectively. From Raman spectroscopy it has been identified that M-DNA is partially transformed to A form owing to mutations and N-DNA on binding of drugs too undergoes transition to A form of DNA. A correlation of thermodynamic contribution and structural data reveal the presence of different binding events in drug and DNA interactions. These events are assumed to be representative of minor groove complexation, reorientation of the drug in the complex, DNA deformation to accommodate the drugs and finally intercalation. Dynamic light scattering and zeta potential data also support differences in structure and mode of binding of N and M DNA. This study highlights that mutations can manifest structural changes in DNA, which may influence the binding efficacy of the drugs. New generation of drugs can be designed which recognize the difference in DNA structure in the cancerous cells instead of their biochemical manifestation.

## Introduction

Improvement in therapeutic activity and selectivity is a major goal in the development of anticancer agents. The genetic differences between the normal cells and cancerous cells are exploited by several molecular targeted drugs like imatinib and trastuzumab, which show promising therapeutic activity and low toxic side effects [1], [2]. Current gene targeting therapeutic strategies still face significant challenges owing to acquired drug resistance and genomic instability of cancer cells [3]–[5]. Targeting the unique biochemical alterations in cancer cells might be feasible approach like increased aerobic glycolysis, oxidative stress etc. The multiple genetic alterations (mutations) disturb the DNA structure in cancer cells and can also be considered as therapeutic target instead of biochemical manifestations. Such alterations in DNA structure were identified in myeloid leukemic cells (K562), where a partial conformational change from B to A form was reported by us [6].

**Figure 1:**
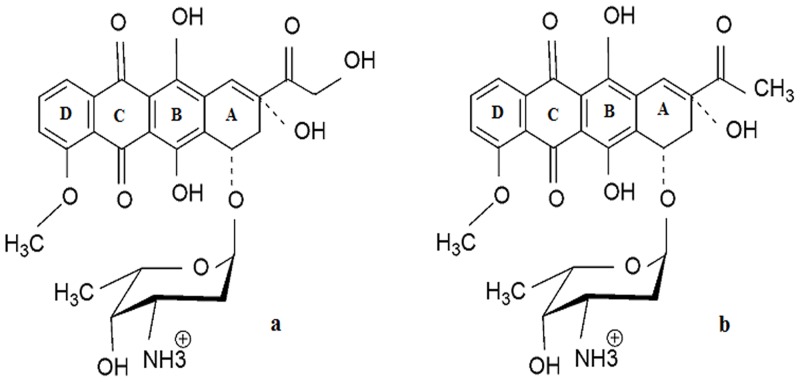
Structural representations of the drugs. Chemical structure of (a) Adriamycin (ADR) and (b) Daunomycin (DNM).

Acute myeloid leukemia is a highly malignant hematopoietic tumor treated by anthracycline antibiotics like adriamycin (ADR) and daunomycin (DNM). These drugs inhibit DNA topoisomerases and subsequently block DNA replication leading to cell death [7]–[9]. The intercalation site of daunomycin is sequence specific, identified as dCpGpATpCpG [10]–[12] According to X-ray crystallographic studies upon intercalation the aglycone chromophore of the drug is inserted into two consecutive base pairs at right angles to the long dimension of DNA and the daunosamine stays in the minor groove (Fig. 1) [13]. The mutated K562 DNA (M-DNA) has been sequenced and ten genes have been identified with acquired mutations when compared with its normal counterpart (N-DNA) [14]. These mutations influence the conformation of the DNA and make ADR and DNM binding more effective compared to normal cells. The changes in structure as diagnosed by circular dichroism (CD) and Fourier transform infrared (FTIR) spectroscopy are attributed to these mutations [6], [15]. Such conformational changes and their influence on binding affinities can be thermodynamically characterized. The present study is an effort in this direction where structural changes are also countenanced by Raman spectroscopy and dynamic light scattering (DLS) studies.

Thermodynamics provides an essential complement to structural studies, which on their own are incapable of defining molecular forces that govern complex formation. There exist a number of thermodynamic studies addressing changes in free energy on intercalation of different drugs and their variants [16]–[19]. From such studies it is highlighted that intercalation is not achieved in one step, rather it gains stability after various structural orientations of the drug as well as DNA. According to Chaires et al. intercalation of the anthracycline drug is achieved by outside binding followed by intercalation and reshuffling of the drug in the intercalation site; this is similar to theoretical predictions of Wilhelm et al [20], [21]. Others like Rizzzo et al. have proposed five step kinetic modes [22]. Raman and vibrational spectroscopy have been widely used as a major tool to characterize the nature of drug-DNA complexation and effect of such interactions in transition of the secondary structure of nucleic acids from B to A form [23]–[25]. DLS is convenient and efficient method of determining size of biologically important macromolecules [26], [27]. This technique was used to measure DNA size transition on formation of drug-DNA complexes. Effective charge density is a crucial parameter in determining the structure and morphology of DNA in complexed state and this was expressed by zeta potential [27]. Lacunae still exists in correlation of thermodynamic data with structural changes and charge neutralization due to complexation and this has been addressed in the present study.

## Experimental Procedures

### Ethical Statement

Collection of human blood samples from healthy donors was approved by Institutional Ethics Committee of West Bengal University of Technology and informed written consent has been taken from each person to satisfy the ethical concerns.

### Preparation of DNA and drug solutions

Genomic DNA was isolated from normal human blood collected from healthy voluntary donors and also from myeloid leukemia cell line K562 using QIAmp Blood Midi Kit purchased from QIAGEN (Hilden, Germany). Isolated DNA was further purified by phenol chloroform method and lyophilized [6], [15]. Lyophilized DNA was dissolved in 50 mM phosphate buffer (pH 6.5) when required and the purity was ascertained by the ratio of A_260_/A_280_; samples with the ratio between of 1.8–2.0 were considered pure. Concentration of DNA was determined by the absorption at 260 nm and the molarity (base pairs) was calculated based on ε_260_ = 13,200 M^−1^ cm^−1^. Adriamycin and daunomycin were purchased from Sigma-Aldrich Chemicals Company (St. Louis, MO, USA). The stock solutions of the drugs were prepared in 50 mM phosphate buffer (pH 6.5) and 5% DMSO was used as and when required. Stock solutions were further diluted by the same buffer to the experimentally required concentrations and stored at 4°C. All other reagents used were of analytical reagent grade. All the buffer solutions were prepared in MilliQ water.

### Isothermal Titration Calorimetry

DNA intercalators like ADR and DNM containing planar aromatic chromophores require higher concentrations for ITC measurements. At the high concentrations they exhibit either aggregation or self-association impeding ITC performance. Such aggregation or self assembly has been reported for molecules like thiazotropsin and DNA was evidenced by ITC on dilution [28]. To overcome this problem in the present experiments drugs at low concentrations were loaded in the cell and DNA was injected into it from the syringe (termed as Reverse-ITC experiment) [29]. Calorimetric titrations were performed at 25°C in a MicroCal VP-ITC microcalorimeter. Before loading, the solutions were thoroughly degassed. DNA samples in 50 mM Phosphate buffer (pH 6.5) were used in all the experiments. Typically, 200 µl drug solution (3.5 µM ADR/DNM for M-DNA and 0.17 µM ADR/DNM for N-DNA), loaded in the calorimetric cell was titrated against 0.2 mM of each DNA solution separately (20 injections of 2 µl each). Sequential titrations were performed to ensure full occupancy of the binding sites by loading and titrating with the same ligand without removing the samples from the cell until the titration signal was essentially constant as described by Arora et al. [30] Each injection generated a heat-burst curve (μcal s^−1^) versus time (min). The area under each peak was determined by integration using Origin software (Microcal, Inc.) to give the measure of the heat associated with the injection. The resulting associated temperatures were plotted against molar ratio. The resulting experimental binding isotherm was corrected for the heat effect of titrating each DNA into buffer. The resulting thermograms did not fit well with one or two site binding models. Sequential binding sites model (Levenberg-Marquardt non-linear least squares curve fitting algorithm) inbuilt in the MicroCal LLC software fitted the best to give association constants (K) and the binding enthalpy (ΔH). Consequently, the changes in Gibbs free energy (ΔG) and the changes in entropy (ΔS) can be calculated using the following equations:

ΔG = −RT lnK and ΔG = ΔH−TΔS

where T is the reaction temperature (in K) and R signifies the universal gas constant (1.986 cal K^−1^ mol^−1^).

### Raman Spectroscopy

Raman spectroscopy is well-established, albeit yet underappreciated method suitable for structural studies of nucleic acids conformations [31], [32]. Drug-DNA complexes were prepared by mixing 2 µM DNA solutions with equimolar concentrations of drugs at 25°C and incubating for 2 hrs. Subsequently Raman spectra of both types of DNA and their respective drug complexes were recorded on a Perkin Elmer Raman spectrometer, using 514.4 nm line of an argon laser. Raman spectra of the two forms of DNA were recorded over the spectral range 400–4000 cm^−1^. The spectra were typically recorded at 4 cm^−1^ slit width with a 2 sec integration time at each 2 cm^−1^ frequency increment. They were routinely background-corrected by subtracting appropriate polynominal function from original curve.

### Dynamic Light Scattering (DLS)

DLS is used to determine the distribution profile of biological macromolecules like proteins, DNA, chromatins etc [33],[34]. In order to study the effect of ADR and DNM on the hydrodynamic size and zeta potential of N-DNA and M-DNA, the samples (2 µM) were treated with equimolar concentration of drugs at 25°C and incubated for 2 hrs. Subsequently these samples were monitored by DLS using a Zetasizer, Nano-ZS instrument (Malvern Instruments, Southborough, UK). Measured size of DNA and their drug complexes were presented as the average value of 100 runs. Nano-ZS (Malvern Instruments, Southborough, UK) using laser Doppler velocimetry and phase analysis light scattering was used for zeta potential measurement. Zeta-potential measurements for different types of DNA and drug-DNA complexes were carried out in the standard capillary electrophoresis cell of the Zetasizer 2000 HS (Malvern, UK). Average values were calculated from three sets of experimental data.

## Results

### Calorimetric studies

**Figure 2:**
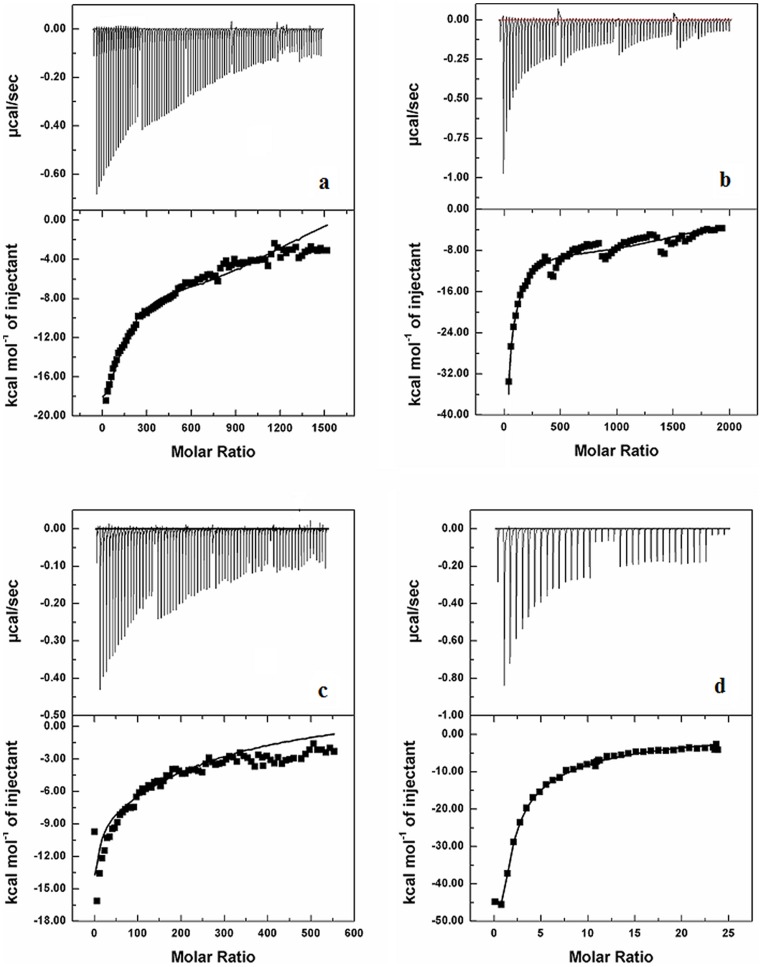
Isothermal calorimetric titrations of drugs with DNA. Thermograms for the sequential titration of 0.2-DNA into 3.5 µM of (a) ADR, (b) DNM and 0.2 mM M-DNA into 0.17 µM of (c) ADR, (d) DNM in 50 mM phosphate buffer (pH 6.5) at 25°C.

Isothermal titration calorimetry (ITC) was used to efficiently characterize and recognize both high affinity and low affinity intermolecular interactions quickly and accurately. Typical experimental ITC thermogram obtained on titration of drugs (ADR/DNM) with N-DNA and M-DNA is illustrated in Fig. 2 and the resulting binding parameters are tabulated in Table 1. For both the drugs examined the ITC thermograms showed negative heat deflection, consistent with exothermic binding to both types of DNA. These thermograms from the built in software fitted best with sequential binding model. Existence of such sequential events could be indentified due to the reverse titration technique where very low concentration of drug is used to overcome self aggregation of drugs. This model yielded four thermodynamically different events of drug N-DNA interaction which are being formed from the other constantly by breaking and making of strong or weak bonds. These events represent different associations between drug and N-DNA (weak or strong association). The binding constants and the thermodynamic parameters associated with each event are tabulated in Table 1. From the Table 1 is observed that N and M DNA interactions with drugs are defined by four and three events, respectively. The extra event in N-DNA-drug interaction is characterized by lowest binding affinity with ADR/DNM of 6.0 E2/9.7 E2 M^−1^ accompanied by positive values of ΔH (6.6 E8/9.1 E7 cal/mol) and ΔS (2.0 E6/3.0 E5 cal/mol/K) (Fig. 2a–b). Highest binding affinities are associated with M-DNA-DNM interactions, consistent with our earlier findings [6].

**Table 1:** Summary of thermodynamic parameters obtained on analysis of ITC thermograms representative of N-DNA and M-DNA interaction with ADR/DNM.

Sample	K_a_ (M^−1^)	ΔH (cal/mol)	ΔS (cal/mol/K)	ΔG (cal/mol)
ADR-Normal DNA	2.5 E4	−4.3±0.18 E6	−4.3 E6	−6.0 E3
	3.0 E3	−3.5±0.11 E7	−1.2 E5	−4.7 E3
	6.0 E2	+6.6±0.27 E8	+2.0 E6	−3.8 E3
	3.7 E3	−2.7±0.1 E9	−9.0 E6	−4.9 E3
DNM-Normal DNA	8.1 E5	−1.7±0.06 E6	−5.8 E3	−8.0 E3
	1.8 E4	−1.6±0.07 E7	−5.3 E4	−5.8 E3
	9.7 E2	+9.1±0.32 E7	+3.0 E5	−4.0 E3
	4.4 E3	−6.6±0.29 E8	−2.2 E6	−5.0 E3
ADR-K562 DNA	1.3 E5	−3.1±0.11 E5	−1.0 E3	−7.0 E3
	3.4 E4	−5.3±0.19 E5	−1.8 E3	−6.2 E3
	2.4 E3	−1.1±0.04 E7	−3.6 E4	−4.6 E3
DNM-K562 DNA	1.2 E9	−4.5±0.17 E4	−109.4	−12.4 E3
	4.6 E5	−6.3±0.26 E4	−185.0	−7.7 E3
	2.0 E4	−3.1±0.11 E5	−1.0 E3	−5.8 E3

### Raman spectroscopy

**Figure 3:**
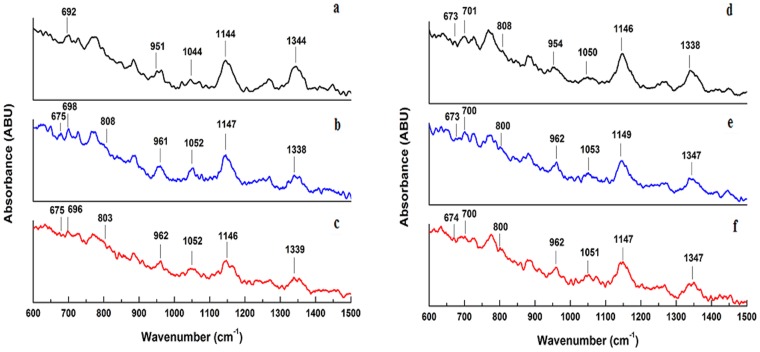
Raman spectra of drug-DNA complexes. Smoothed Raman spectra of (a) 2 µM native N-DNA and its complexes with equimolar (b) ADR and (c) DNM; (d) 2 µM native M-DNA and its complexes with equimolar (e) ADR and (f) DNM in 50 mM phosphate buffer (pH 6.5) at 25°C.

Raman spectroscopy has very high chemical specificity and is routinely used to differentiate between different polymorphs of the same compound. Raman lines near 682, 668, or 625 cm^−1^ representing B (C2′-endo, anti), A (C3′-endo, anti) or Z (C3′-endo, syn) structures respectively, are the most useful for quantitative analysis [35]. The major Raman bands representative of bases (adenine, guanine, cytosine, thymine), deoxyribose and phosphate stretching are listed in Table 2 [36], [37]. The B form DNA is characterized by C2′-*endo* sugar pucker (692 cm^−1^), C2′-*anti* glycosyl torsion (728 cm^−1^), and C2′-H2 (1411 cm^−1^) [24], [36], [38]. It also has characteristic phosphodiester torsions (828 cm^−1^) and phosphodiester O-P-O stretching (1091 cm^−1^) [38], [39]. In the Raman spectra of N/M-DNA and their respective ADR/DNM complexes (Fig. 3a–f), all the above B DNA diagnostic peaks have been identified (some are marked in the figure) and their shifts in drug-DNA complexes have also been recorded and summarized in Table 2. The key structural markers of B DNA identified in N-DNA support that the backbone structure of the DNA remained orderly in B form. In the spectra of N-DNA and ADR/DNM complexes shoulder line appeared at 673 cm^−1^ and 803 cm^−1^, which are marker bands of A DNA related to C3′-endo, anti along with the stretching vibration of O-P-O phosphodiester pucker and glycosyltorsion [40] From these peaks in N-DNA and drug complexes, a partial transition to A form is recognized. These two marker bands of A DNA were also traced in the spectra of M-DNA (Fig. 3d) owing to the mutations, with little shifts in the drug complexes (Fig. 3e–f). These findings are consistent with our previous findings [6].

**Table 2:** Assignment of some of the observed Raman bands in N-DNA/M-DNA and their shifts on ADR/DNM binding.

Assignment	Normal DNA	Normal DNA-ADR	Normal DNA-DNM	K562 DNA	K562 DNA-ADR	K562 DNA-DNM
	Raman Peaks (cm^−1^)					
Ring mode (T)/T breathing vibration	635	633	634	636	634	635
Ring breathing (G) influenced by C2′ endo sugar pucker	692	698	696	701	700	700
Ring breathing (C)	776	768	770	768	763	765
Ring mode (G, A)	1344	1338	1339	1338	1347	1347
Deoxyribose/O-P-O stretching	884	887	889	880	885	885
Deoxyribose	951	961	956	954	962	961
C = O stretching/PO_2_^−^ symmetric stretching	1044	1052	1052	1050	1053	1051
Deoxyribose-phosphate stretching	1144	1147	1146	1146	1149	1147
Change in A DNA markers:						
Ring breathing (G) influenced by C3′ endo sugar pucker	-	675	675	673	673	674
O-P-O stretching vibration	-	808	803	808	800	800

The respective nucleic acid Raman vibrations have also been identified in the Raman spectra and their shifts recorded and reported in Table 2. Higher involvement of guanine, cytosine and ring mode G, A in N-DNA is supported by higher shifts in Raman lines compared to M-DNA. Higher shifts for deoxyribose (951 cm^−1^) are recorded for N- DNA suggesting more backbone conformational changes upon intercalation and similar shifts in M-DNA are due to close vicinity of the drugs to phosphate groups involved in external binding. Other bands representative of phosphate deoxyribose stretching also show similar trends as illustrated in Table 2. The results suggest higher intercalation in B form of N-DNA and higher external binding in A form of M-DNA.

### Hydrodynamic characterization of drug-DNA interaction

**Figure 4:**
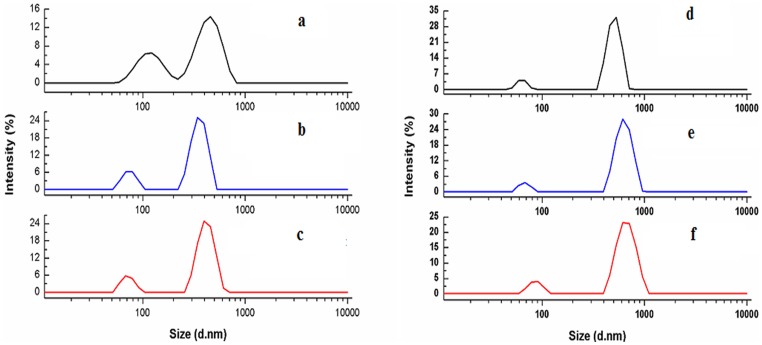
Dynamic Light Scattering (DLS) profile of drug-DNA complexes. Intensity weighted distribution functions of (a) 2 µM native N-DNA and its complexes with equimolar (b) ADR and (c) DNM; (d) 2 µM native M-DNA and its complexes with equimolar (e) ADR and (f) DNM in 50 mM phosphate buffer (pH 6.5) at 25°C.

Dynamic light scattering (DLS) has been employed to study the size transition of N/M DNA on formation of complexes with ADR/DNM in solution. The drugs-N-DNA complexes exhibited reduced size compared to free DNA. The Z_av_ diameter of N-DNA decreased from 877.7 nm to 649.4 nm and 783.1 nm for ADR and DNM complexes respectively (Fig. 4a–c). The Z_av_ diameter of M-DNA was observed to be much lower (457.2 nm) than N-DNA and its complexes with drugs recorded significant increase in size to 586.1 nm and 588.9 nm for ADR and DNM respectively (Fig. 4d–f). Here it is observed that mutation induced partial transition to A form is more compact than B form DNA. N-DNA on complexation with ADR/DNM undergoes partial B to A transition resulting in reduced size. M-DNA on the other hand favours external binding leading to increase in size.

**Figure 5:**
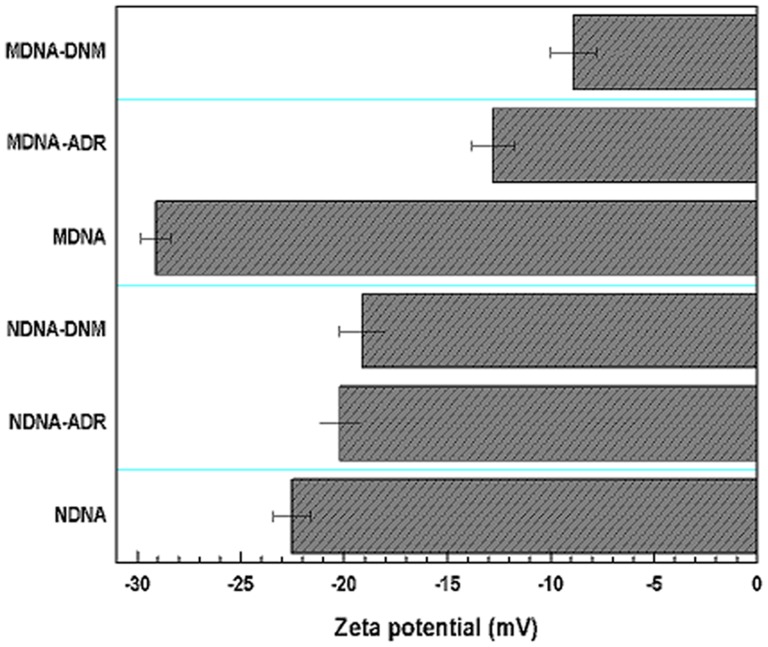
Zeta potential measurements of drug-DNA complexes. Zeta potential of 2 µM native N-DNA and M-DNA and their complexes containing equimolar drugs in 50 mM phosphate buffer (pH 6.5) at 25°C. Error bars indicate the standard error (SE) for N = 3 independent experiments.

Zeta potential is a measure of the surface electrical charge of the particles. Values of the zeta-potential of drug/DNA indirectly reflect net surface charge of the complexes and can therefore be used to evaluate the extent of interaction of the drug with DNA [41]. Measurement of zeta potential of ropinirole hydrochloride and aspirin complexes with human holo-transferrin revealed existence of electrostatic and hydrophobic interactions in the complex [42]. These charges and in turn the binding forces are expected to be modulated in drug-DNA complexes hence their zeta potential values were determined along with their respective free forms. The recorded zeta potentials of N and M DNA were −22.5±0.9 mV and −29.1±0.7 mV respectively (Fig. 5). From these values it is inferred that in M-DNA the phosphate groups carrying negative charge are more exposed than in N-DNA. This is attributed to the conformational difference in N-DNA and M-DNA, where M-DNA adopts a partial transition to A form of DNA resulting in larger turns and exposure of more phosphate groups. Formation of N-DNA, complexes of ADR and DNM resulted in increase in potential to −20.2±1.1 mV and −19.1±1.0 mV respectively. In M-DNA-drug complexes the potential increased to −12.8±1.2 mV and −8.9±1.3 mV for ADR for DNM, respectively (Fig. 5). The higher increase in zeta potential of M-DNA confirmed that electrostatic forces (backbone binding) were major binding forces with contribution from hydrophobic forces (intercalation). Less increase of the same in N-DNA suggests that electrostatic interaction exists but hydrophobic interactions like intercalation are dominant binding forces.

## Discussion

**Figure 6:**
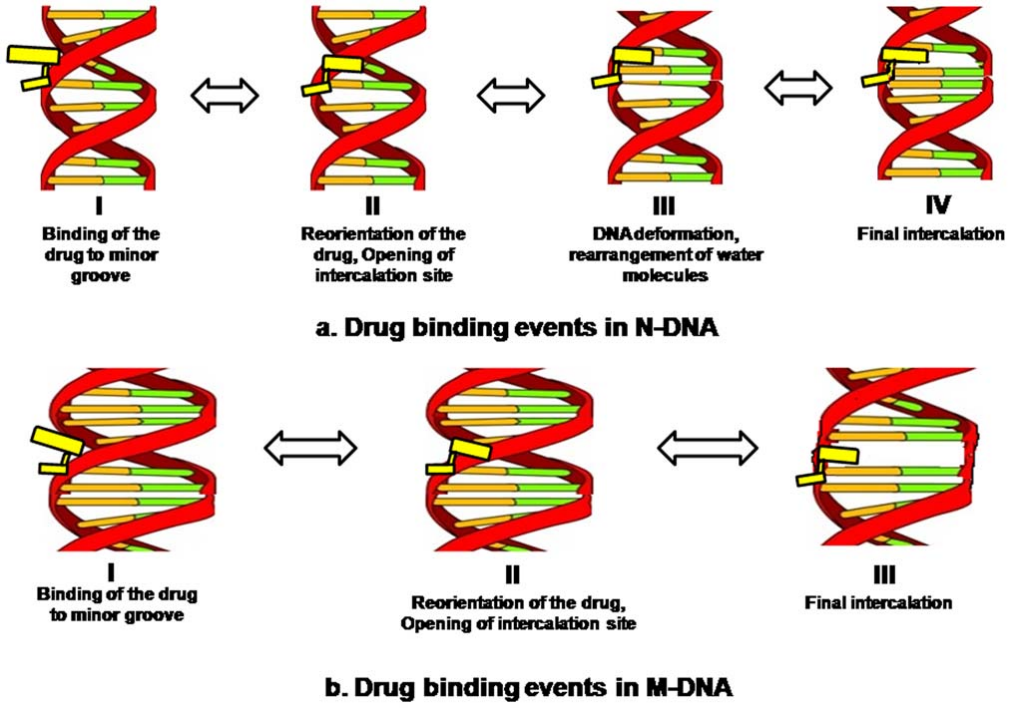
Schematic representation of drug DNA interaction. Possible events taking place during interaction of (a) N-DNA and (b) M-DNA with ADR/DNM.

The functional consequences of mutations in cancer genome and their possible affinity to anticancer drugs compared to normal genome have great implication in rational designing or modifying drugs and are needed to be explored. The cognizance of correct experimental design and selection of suitable binding models cannot be overlooked. ITC is a powerful technique for the accurate and precise measurement of the affinity of biomolecular interactions and defines binding mechanism and has application in rational drug designing. It is important to realize that ΔG and its ΔH and ΔS constituents depend upon differences between free and bound states for both of the interacting partners (drug and DNA). The ITC thermograms shown in Fig. 2a–b represent heat exchange upon titration of ADR and DNM respectively with N-DNA. These thermograms for N-DNA and ADR/DNM interaction on global analysis using sequential model evidence four possible associations between DNA and drug and each can be distinguished by representative binding constants and thermodynamic components (Table 1). The M-DNA drug titration thermograms (Fig 2c–d) are representative of three associations. Both N/M DNA-drug interactions are associated with three states which are characterized by -ve ΔH and -ve ΔS, diagnosing formation of strong associations with high binding affinities. These associations can be perceived as drug bound to the DNA minor grooves and subsequent reorientation of the drug in the complex and intercalation which are stabilized predominantly by van der Waals forces and hydrogen bonds as illustrated in Fig. 6. The distinctive +ve ΔH and +ve ΔS state in N-DNA is an enthalpically unfavorable process, driven by entropy. In such processes bonds are broken to bring higher disorder or openness (B to A transition) in the structure in order to accommodate a rather complex intercalation with bulky group binding in the minor groove with release of water from binding interface to the bulk and stabilized by hydrophobic forces [13], [43]. This state (+ve ΔH and +ve ΔS) is not observed in M-DNA as the DNA adapts partial B to A transition due to mutation and further intercalation does not bring about significant conformational changes. These structural changes are evidenced by appearance of new peaks in Raman spectra in N-DNA drug complexes, which are representative of A form DNA (Table 2). M-DNA in native form itself has signatures of A form DNA which has also been consolidated earlier by FTIR and CD [6]. Existing literature on drug DNA interaction theoretical predictions have been made of existence of other forms of associations before final intercalation [44]. Since no covalent bonds are formed during these interactions it is assumed here that all forms exist in equilibrium and the equilibrium is shifted by external conditions.

In the process of intercalation, change in the unwinding angles of the two base pairs distorts the backbone resulting in a compressed DNA structure as affirmed by DLS findings. The drug complexed N-DNA has reduced size due to transition from B to A upon intercalation which makes the helix skewer and distorted. There is an increase in size for drug-M-DNA complexes due to external binding influenced by backbone exposure in A DNA, supported by increase in the zeta potential due to neutralization of charge in the drug complexes (Fig. 4–5). Less increase in zeta potential in N DNA-drug complexes suggest that intercalation is more favorable mode of binding eliminating excess backbone binding.

## Conclusion

Results from all the three techniques are culminating to opine that structural difference exists between N-DNA and M-DNA, which is recognized by the drugs ADR/DNM. This difference can be exploited in designing more specific drugs for cancer cells. In complexes of N-DNA and DNM a decrease in B-form DNA structure in favor of A-form DNA was observed. It is suggested that stacking force and the hydrogen bond between base pairs were destructed and a part of B-form DNA became single-stranded. Further the size and charge compensation of drug-DNA complexes espouse the above analogy. The application of sequential binding model is most favorable and sheds light on undergoing mechanistic states in drug-DNA interaction. The thermodynamic analysis is compatible with earlier theoretical predictions. These results explain higher toxicity of ADR/DNM in K562 cells compared to normal cells [15] and emphasize the need to design drugs which can selectively recognize DNA conformational changes in cancerous cells resulting in increased therapeutic ratio of the drugs. Further studies involving structural changes in chromatin of cancerous cells are in progress to evaluate these findings for application in cancer therapy.
